# Description of the first species of Glomeridesmida from Thailand (Diplopoda, Glomeridesmida, Glomeridesmidae)

**DOI:** 10.3897/zookeys.1024.63678

**Published:** 2021-03-16

**Authors:** Thomas Wesener, Nattarin Wongthamwanich, Leif Moritz

**Affiliations:** 1 Zoological Research Museum Alexander Koenig, Leibniz Institute for Animal Biodiversity, Adenauerallee 160, 53113 Bonn, Germany Leibniz Institute for Animal Biodiversity Bonn Germany; 2 Department of Biology, Faculty of Science, Srinakharinwirot University, Bangkok 10110, Thailand Srinakharinwirot University Bangkok Thailand; 3 University of Bonn, Institute for Evolutionary Biology and Ecology, An der Immenburg 1, 53121 Bonn, Germany University of Bonn Bonn Germany

**Keywords:** Biodiversity, Krabi, limestone hill, soil arthropod, Southeast Asia

## Abstract

With three genera and 35 previously known species from India, SE Asia, Central and South America, Glomeridesmida are one of the least diverse Diplopoda groups. Here we describe *Glomeridesmus siamensis***sp. nov.**, the first species of the order Glomeridesmida from Thailand. The geographically nearest confamiliar species have been described from southern India, Sumatra and Java. The species is described combining photographs, light- and scanning electron microscopy of mature and younger males, females and juveniles. Several characters are illustrated for the first time for an Asian representative of the family Glomeridesmidae. In addition to the type locality of *G. siamensis***sp. nov.** from Krabi province, locality data of unidentified *Glomeridesmus* from Thailand are also given. These data are providing further evidence that the Glomeridesmida are not uncommon, but overlooked as they are small and difficult to collect. The unusual telopods and other morphological characters of *G. siamensis***sp. nov.** differ considerably from the few *Glomeridesmus* males described from Central and South America as well as from India, but the unclear status of two generic names available for species from Indonesia prevents us from adding another generic name to this small and understudied order.

## Introduction

The third smallest millipede order (Diplopoda) in terms of diversity is the Glomeridesmida with 35 described species in three genera (Jeekel 2003; Iniesta et al. 2012; Enghoff et al. 2015; Mauriès 2020). The Glomeridesmida are currently classified as the basalmost order within the Pentazonia (Blanke and Wesener 2014), which also includes the volvatory (rolling-up) millipede orders Glomerida and Sphaerotheriida, but might actually be the sister-group to the Holarctic pill millipedes, the Glomerida (Oeyen and Wesener 2018). The Glomeridesmida is divided into two families (Enghoff et al. 2015), the obligate termitophilous Termitodesmidae (5 species) and the Glomeridesmidae (30 species). While the Termitodesmidae are restricted to India and continental Southeast Asia (Malaysia and Vietnam), the Glomeridesmidae show a much wider and disjunct distribution with representatives in the Caribbean, Central America and the northern half of South America, India, Southeast Asia (Shelley and Golovatch 2011) and Oceania (Shelley 2011). No described *Glomeridesmus* species is known from the Southeast Asian states of Thailand (Enghoff 2005), Laos (Likhitrakarn et al. 2014), Cambodia (Likhitrakarn et al. 2015) or Myanmar (Likhitrakarn et al. 2017). As recently shown, Glomeridesmidae are not rare in Javan rainforests (Hilgert et al. 2019).

The unusual appearance of the Glomeridesmida might partly resemble the ground-plan of chilognathan Diplopoda (Enghoff 1990). While the first species of a glomeridesmid was described in 1844 (Gervais), the first male specimens were not encountered until almost 100 years later (Loomis 1936) and still needed another five years to be recognized as such (Carl 1941). Until then, the exceptionally large and conspicuous female ovipositors were interpreted as male ‘penes’. However, either live observations, or even male specimens of Glomeridesmida are still unknown for nearly all species or very rare (Mauriès 1980, 2020; Iniesta et al. 2012). The male telopods (terminal legs modified for mating) provide the most important taxonomic characters in pentazonian millipedes at the generic level (Mauriès 2005; Wesener and VandenSpiegel 2009; Oeyen and Wesener 2018). However, telopod characters are still only known from nine of the 35 glomeridesmid species (Jeekel 2003). Only five species have been extensively described, based on numerous specimens, and thus allowing for detailed comparisons to be made: a single species each from India (Carl 1942), Central America (Mauriès 1980) and a cave in Brazil (Iniesta et al. 2012), and two species recently described from French Guiana (Mauriès 2020). However, males are still undescribed for SE Asian glomeridesmids. Reliable taxonomic characters of the Glomeridesmidae on the species- or generic-level, aside from habitus, body size and colour pattern, have not been established yet. Therefore, we document as many potential taxonomic characters as possible to allow future comparisons with other Glomeridesmida species. The first representative of the group from Thailand is described here based on male and female specimens: somatic as well as sexual characters are described in detail.

## Material and methods

### Abbreviations

**NHMD**Natural History Museum of Denmark, University of Copenhagen;

**SEM** scanning electron microscopy;

**ZCSWU** Zoological Collection of Srinakharinwirot University, Bangkok, Thailand;

**ZFMK**Zoological Research Museum A. Koenig, Leibniz Institute for Animal Biodiversity, Bonn, Germany;

**ZFMK-MYR** collection number of the Myriapoda collection at the ZFMK.

### Preparation, illustrations and terminology

Specimens were preserved in 95% ethanol and are stored in the collections of the ZCSWU and ZFMK. Dissections and examinations were done using an Olympus SZX12 stereo-microscope. For scanning electron microscopy, the samples were dehydrated via an ethanol series, mounted with conductive tape on a stub and dried overnight. The stub was sputter-coated with gold with a Cressington 108 auto sputter coater. Images were obtained using a Zeiss Sigma 300 VP SEM. Multi-layer photographs were taken with a Leica Z6 Imaging-System based at the ZFMK. Stacked images were put together using the Zerene Stacker version 1.04. All images were later modified using Gimp 2.10 and assembled into plates using Inkscape 1.0.1.

Terminology of morphological characters follows Iniesta et al. (2012), for other morphological characters the terminology of Sphaerotheriida (Wesener 2016) and Glomerida (Oeyen and Wesener 2018) was utilized. Usually in pentazonians (e.g. Wesener 2016), specimens with an incomplete number of segments and/or leg pairs are defined as juveniles, while specimens with the full number of segments and legs, but immature secondary sexual characters, are named immatures. This distinction into juveniles and immatures is difficult in Glomeridesmida whose development is only rudimentarily known (Enghoff et al. 1993; Mauriès 2020). Here, we call specimens without the full complement of leg pairs or segments, but with visible ovipositors or telopods immatures and smaller specimens without visible secondary sexual characters juveniles. Additional specimens of undescribed species are housed in the collection of the NHMD.

## Results

### Taxonomy

#### Superorder Limacomorpha Pocock, 1894

**Order Glomeridesmida Latzel, 1884**

**Family Glomeridesmidae Latzel, 1884**

##### Zephroniodesmidae

Taxon classificationAnimaliaGlomeridesmidaGlomeridesmidae

Cook, 1895

C7EF9E4E-53E0-5B0C-A349-A233E0EEB8E1

###### Remarks.

The family Glomeridesmidae was partly re-characterized recently (Iniesta et al. 2012; Enghoff et al. 2015).

##### Genus Glomeridesmus

Taxon classificationAnimaliaGlomeridesmidaGlomeridesmidae

Gervais, 1844

8C513418-2504-56A9-8B4A-F3C25AB04739

Glomeridesmus Gervais, 1844a: xxvii. Gervais 1844b: 61; 1847: 86; 1859: 2. Latzel 1884: 59, 124. Pocock 1894a: 332; 1894b: 476. Silvestri 1896: 201; 1898: 645; 1902: 183; 1903: 22. Brölemann 1898: 256. Attems 1928: 209. Verhoeff 1929: 1377. Loomis 1936: 9; 1964: 9; 1968: 7; 1975: 168. Carl 1941: 250; 1942: 134–167. Jeekel 1971: 33; 2003: 103. Shear 1974: 245. Hoffman 1980: 60; 1999: 19. Shelley 2011: 2. Iniesta et al. 2012: 29. Mauriès 1980: 1060; 2020: 51.
Zephroniodesmus Pocock, 1894b: 476. Silvestri 1896: 201; 1898: 645. Attems 1926: 116. Verhoeff 1929: 1377. Loomis 1936: 9. Carl 1942: 165. Jeekel 1971: 33. Synonymized by Loomis (1936).
Javadesmus Verhoeff, 1929: 1377. Carl 1942: 150, 165. Jeekel 1971: 33. Synonymized by Carl (1942)

###### Type species.

*Glomeridesmus porcellus* Gervais & Goudot, 1844 (Colombia)

*Javadesmus*: *Glomeridesmus javanicus* Attems, 1907 (Indonesia: Java)

*Zephroniodesmus*: *Glomeridesmus sumatranus* Pocock, 1894 (Indonesia: Sumatra).

###### Species included.

30, including the species described below (Jeekel 2003; Iniesta et al. 2012; Mauriès 2020).

###### Distribution.

Central America and northern half of South America and the Caribbean (26); India (1); Indonesia (2); Thailand (1 described below). Unidentified specimens from various localities in SE Asia and Oceania (Shelley 2011).

###### Comment.

The position of the new species in the genus *Glomeridesmus* is tentative at best. As two genus names (*Zephroniodesmus*, *Javadesmus*) synonymized with *Glomeridesmus* (Loomis 1936, Carl 1942) are available for SE Asian Glomeridesmidae and were based on female specimens, both require redescriptions based on topotypic male material. Therefore, we hesitate to introduce a third genus name, which might later turn out to be synonymous. The species from Thailand described here differs significantly from the known Indian and American species at least in the telopod morphology, and potentially in other previously undocumented morphological characters.

##### Glomeridesmus siamensis

Taxon classificationAnimaliaGlomeridesmidaGlomeridesmidae

sp. nov.

2AB443F1-DFF4-5118-AE52-60106B9D2694

http://zoobank.org/9FDB569D-C0AC-4133-B271-62554FD2D757

**Figure 1.**
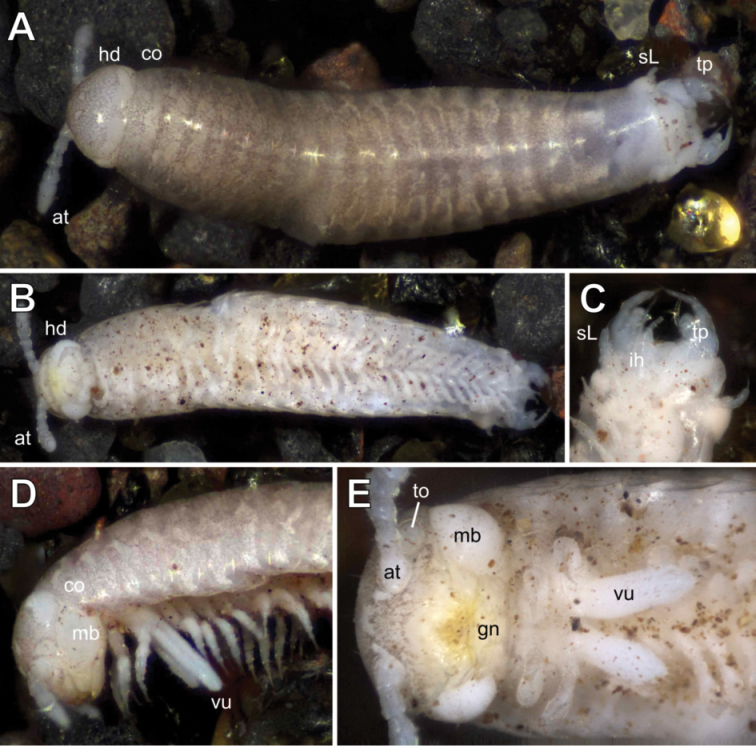
*Glomeridesmus siamensis* sp. nov., photographs **A–C** male holotype (**ZCSWU Myr D000011**) **A** habitus, dorsal view **B** habitus, ventral view **C** telopods, ventral view **D, E** female paratype (**ZFMK MYR10302**) **D** head and anterior body-rings, lateral view **E** head and anterior body-rings, ventral view. Not to scale. **Abbreviations**: at = antennae, co = collum, gn = gnathochilarium, hd = head, ih = inner horns, mb = mandibular base, sL = sensory leg, to = Tömösváry organ, tp = telopod, vu = vulva.

**Figure 2.**
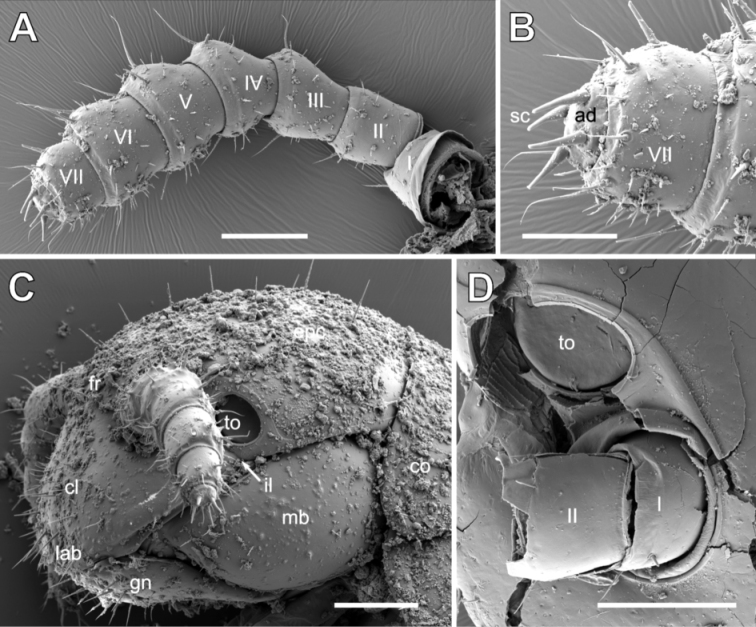
*Glomeridesmus siamensis* sp. nov., head, SEM**A, B, D** male holotype (**ZCSWU Myr D000011**) **A** antennae **B** apical antennomeres **C** juvenile paratype (**ZFMK MYR10304**), head, lateral view **D** antennal base and Tömösváry organ. **Abbreviations**: I – VII = antennomeres, ad = apical disc, cl = clypeus, co = collum, epc = epicranium, fr = frons, gn = gnathochilarium, il = incisura lateralis, lab = labrum, mb = mandibular base, sc = sensory cones, to = Tömösváry organ. Scale bars: 100 µm (**A, C, D**), 50 µm (**B**).

**Figure 3.**
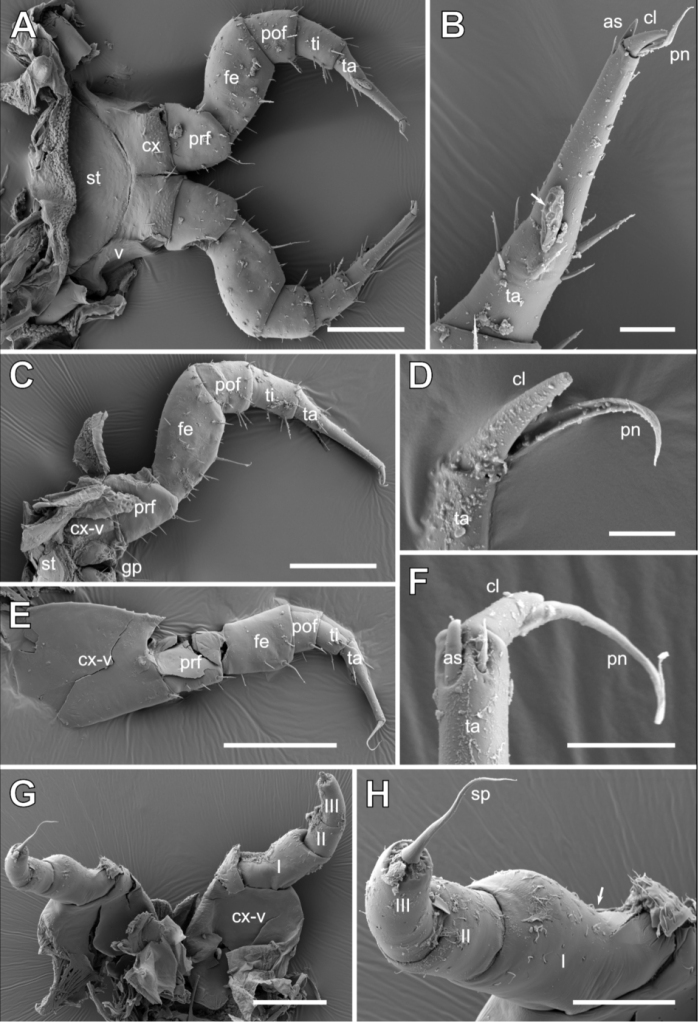
*Glomeridesmus siamensis* sp. nov., male holotype (**ZCSWU Myr D000011**), legs, SEM**A** leg 1, posterior view **B** leg 1, tarsus, arrow indicated parasitic fungi **C** leg 2 with gonopore, posterior view **D** leg 2, tarsus **E** leg 34, posterior view **F** leg 34, tarsus **G** sensory leg (leg 35), posterior view **H** sensory leg (leg 35), detail of podomeres, arrow indicated dent in podomere I. **Abbreviations**: I–III = podomeres, as = apical spine, cl = claw, cx = coxa, fe = femur, gp = gonopore, pn = paronychium, pof = postfemur, prf = prefemur, sp = spine, st = sternite, ta = tarsus, ti = tibia, v = stigmatic plate. Scale bars: 100 µm (**A, C, E, G**), 20 µm (**B**), 10 µm (**D, F**), 50 µm (**H**).

**Figure 4.**
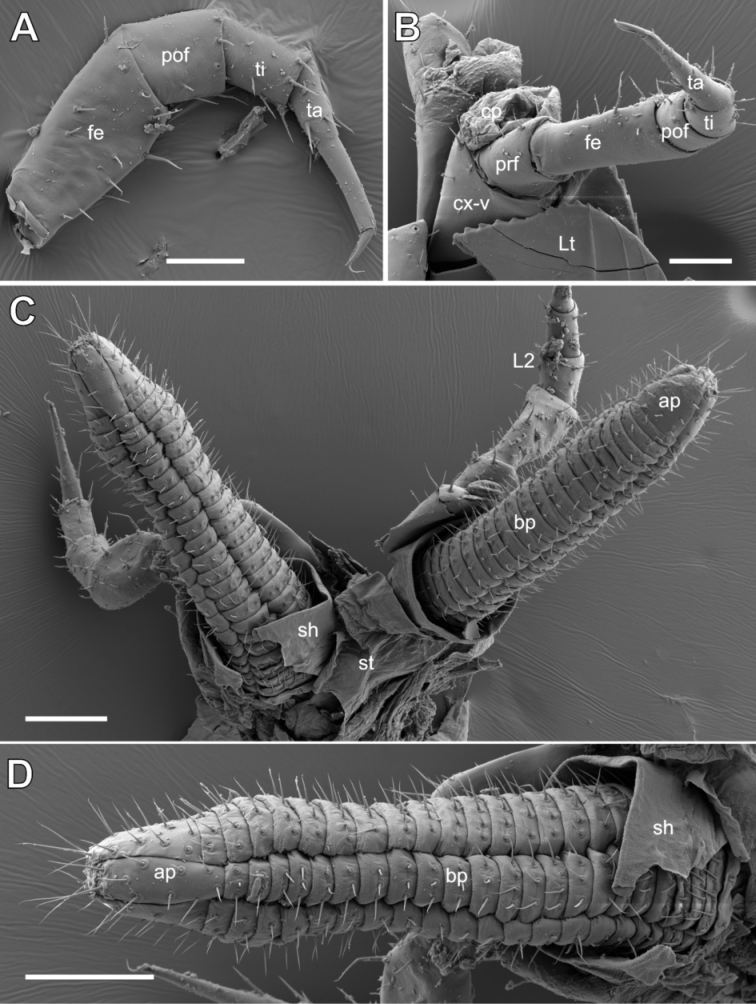
*Glomeridesmus siamensis* sp. nov., female paratype (**ZFMK MYR10302**), legs and ovipositor, SEM**A** leg 23, posterior view **B** leg 23, ventral view **C** leg 2 with ovipositor, ventral view **D** ovipositors, posterior view. **Abbreviations**: ap = apical part of ovipositor, bp = basal part of ovipositor, cp = coxal pouch, cx = coxa, fe = femur, L2 = leg 2, Lt = pleurite, pof = postfemur, prf = prefemur, sh = sheet, st = sternite, ta = tarsus, ti = tibia, v = stigmatic plate. Scale bars: 50 µm (**A, B**), 100 µm (**C, D**).

**Figure 5.**
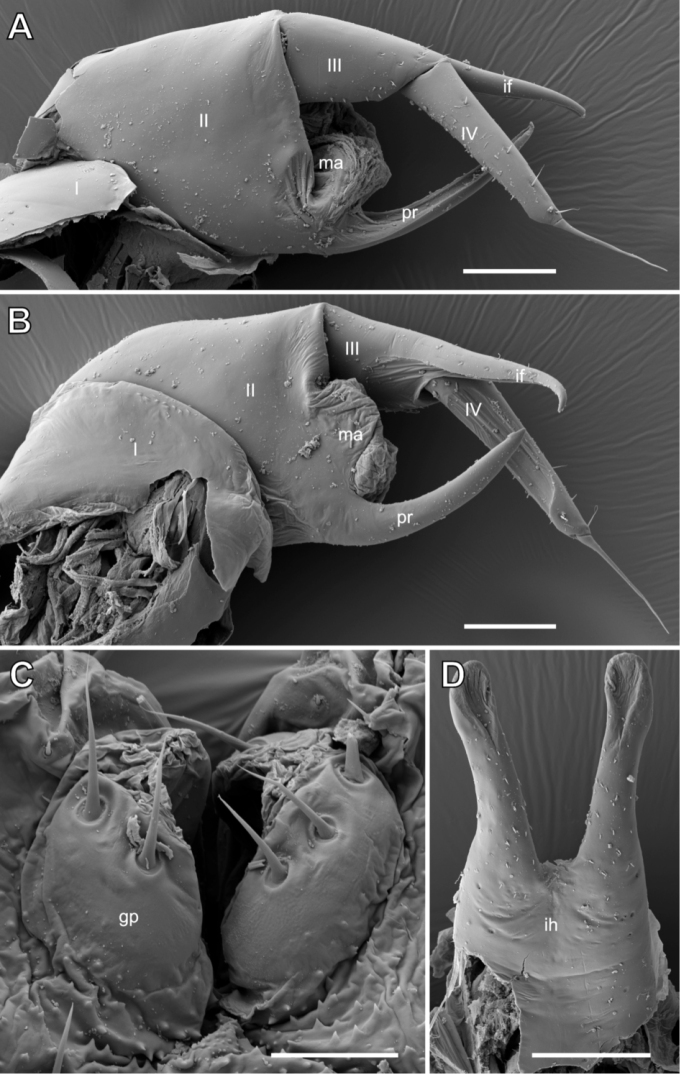
*Glomeridesmus siamensis* sp. nov., male holotype (**ZCSWU Myr D000011**), male sexual characters **A** telopod, anterior view **B** telopod, posterior view **C** gonopore on leg 2, posterior view **D** inner horns, anterior view. **Abbreviations**: I – IV = telopoditomeres, ff = immobile finger, gp = gonopore, ih = inner horns, ma = membranous area, pr = projection. Scale bars: 50 µm (**A, B, D**), 20 µm (**C**).

**Figure 6.**
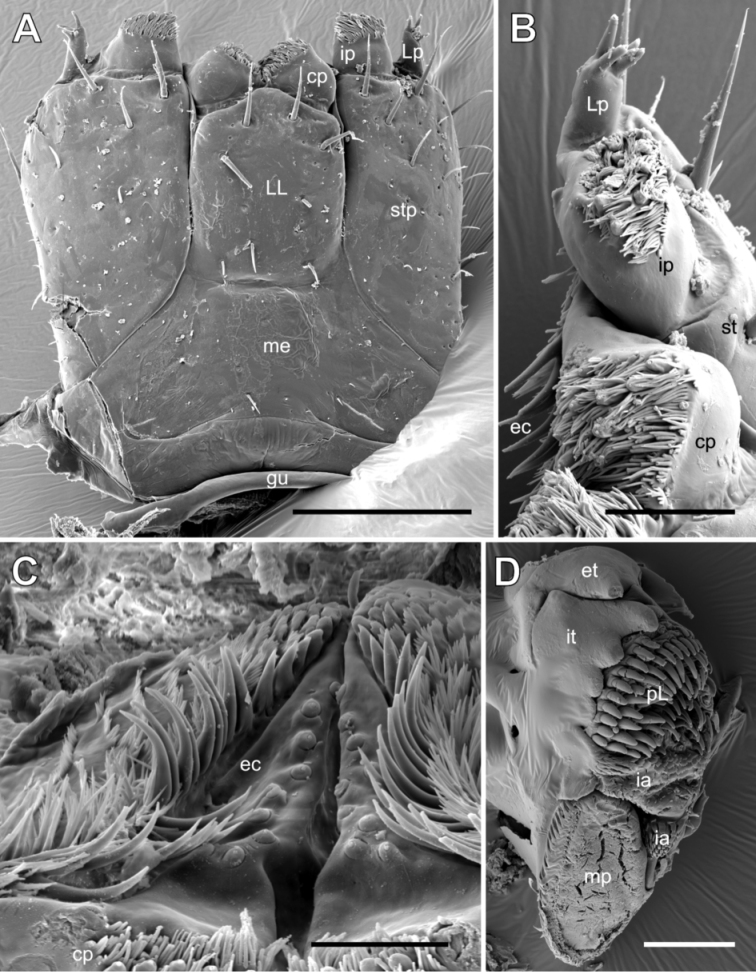
*Glomeridesmus siamensis* sp. nov., male holotype (**ZCSWU Myr D000011**) mouthparts **A** gnathochilarium, ventral view **B** gnathochilarium, palps and central pad, frontal view **C** endochilarium, dorsal view **D** mandibular gnathal lobe, mesal view. **Abbreviations**: cp = central pad, ec = endochilarium, et = external tooth, gu = gula, ip = inner palp, ia = intermediate area, it = inner tooth, LL = lamella lingualis, Lp = lateral palp, me = mentum, mp = molar plate, pL = pectinate lamellae, stp = stipes. Scale bars: 100 µm (**A**), 50 µm (**B, D**), 20 µm (**C**).

**Figure 7.**
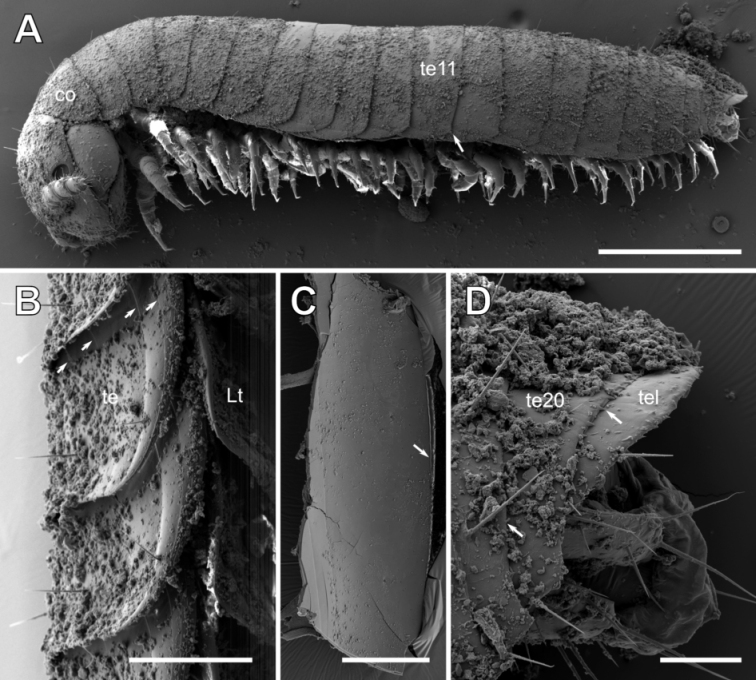
*Glomeridesmus siamensis* sp. nov., body-rings **A, B, D** juvenile paratype **(ZFMK MYR10304**) **A** habitus, lateral view, arrow indicates drawn out lateral tip of tergite **B** detail of tergite margin, latero-ventral view, arrow indicates striae on tergite **C** male holotype (**ZCSWU Myr D000011**), endotergum, arrow indicates posterior margin of tergite **D** posterior body-rings, lateral view, arrows indicate teeth on posterior margin of tergite. **Abbreviations**: co = collum, Lt = pleurite, te = tergite, tel = telson. Scale bars: 500 µm (**A**), 100 µm (**B, C**), 50 µm (**D**).

Figures 1 , 2 , 3 , 4 , 5 , 6 , 7

###### Material examined.

***Holotype*:** 1 M, **ZCSWU Myr D000011 (THAI11)**, Thailand, Krabi Province, N. of Krabi Town, western aspect of Tiger Cave temple (Wat Tham Suea), overgrown rocks next to rubber plantation, 08°07'23.8"N, 098°55'18.9"E. leg. 27.VII.2017, Wesener, Wongthamwanich, Nawanetiwong, Moritz.

***Paratypes*:** 1 immature M, **ZFMK MYR10301**, same data as holotype; 1 F, **ZFMK MYR10302**; 5 juveniles, **ZCSWU Myr D000012–16**; 4 juveniles, **ZFMK MYR10303–10306**, same data as holotype.

###### Etymology.

Siamensis, noun in apposition, after the type locality in Thailand.

###### Diagnosis.

Small (5–7 mm) dark grey glomeridesmid with white legs (Fig. 1A, B). Antennae short (Figs 1A, B, 2A–C). Coxal pouches starting at leg 9, all walking legs with apical spines and paronychium (Figs 3A–F, 4A–C). Last pleurite posterior margin well-rounded in female. Male sensory leg, podomere 1 with a conspicuous excavation on its mesal margin, not known from other *Glomeridesmus* (Fig. 3G, H). Male telopods unique: Inner horns slender, widely separated, a character only shared with *G. indus* Carl, 1942. Telopod, podomere 2 rectangular, with a unique, strongly elongated and slender process protruding between movable (podomere 4) and immovable finger (process of podomere 3) (Fig. 5A, B). Podomere 3 with a slender immovable projection and podomere 4 forming a long movable finger. Immovable finger long and visibly laterally in *G. siamensis* sp. nov. (Figs 1C, 5A, B), in contrast to South American species such as *G. spelaeus* Iniesta & Wesener, 2012 and *G. arcostriatus* Mauriès, 2020, where it is hidden in lateral view. Lamellae linguales of gnathochilarium fused with one another, but with a transverse suture towards mentum, lamellae with two central setae at margin and two lateral setae more posteriorly in *G. siamensis* sp. nov. (Fig. 6A), while all four setae are marginal in *G. spelaeus* (compare Iniesta et al. 2012).

###### Description.

Based on male holotype (**ZCSWU Myr D000011**) and female paratype (**ZFMK MYR10302**).

***Measurements*:** Largest adult female (20+AS tergites, 35+1 leg pairs): length: ~6 mm; width (midbody): 1.0 mm. Male (19+AS tergites, 33+1+T leg pairs, holotype male): length: ~5 mm; width (midbody): 0.9 mm; immature (?) male (19+AS tergites, 32+1+T,): length: ~4 mm, width: 0.8 mm.

***Colour in ethanol*:** Tergites and dorsal side of head grayish-brown (Fig. 1), legs, antennae and ventral side whitish (Fig. 1A, B).

***Head*:** General shape typical for the family (Figs 1D, E, 2C). Epicranium glabrous; frons, clypeus and labrum with several isolated setae (Fig. 2C). Antennae widely separated by a distance slightly longer than twice width of first antennomere (Fig. 1A, E). Antennae and organ of Tömösváry both surrounded by individual cuticular rims, both on an elevated plateau (Fig. 2D). Genae (area below the antennae) almost non-existent, with incisura lateralis. Undivided basal joint of mandible large and visible in dorsal view.

Antennae consisting of seven joints, each covered with numerous setae. Size of joints 1 = 2 = 3 = 4 = 5 > 6 (Fig. 2A). Apical disc carrying four long apical cones; sensilla basiconica apparently absent (Fig. 2B).

Tömösváry organs as large as antennal base, of well-rounded oval shape, interior covered by a sclerotized plate; surrounded by cuticular rim and several very short setae (Fig. 2C, D).

Gnathochilarium with very broad gula (hypostoma). Cardines very small, separated from basal part of gnathochilarium. Proximal fourth of mentum towards gula distinctly elevated, transverse suture visible between mentum and lamellae linguales. Lamellae linguales fused to one another, not separated by a longitudinal suture, apically with four long setae, central pair at apical margin, lateral two setae located more posteriorly. Whole surface of gnathochilarium covered with few, isolated setae. Stipites laterally without sclerotized ledge. Stipites apically with 2 extra-long setae (Fig. 6A). Stipites lateral palpi slender, carrying 4 slender sensory cones; inner palpi very wide, carrying 12–15 long, tube-shaped sensory cones interspersed between long cuticular fibres (Fig. 6B). Central pads (modified central palpi of lamellae linguales) large, mesally pointing towards and touching one another, in ventral view covered with numerous long, tube-shaped sensory cones and long cuticular fibers (Fig. 6B). Basally of central pads on endochilarium with two parallel rows of 9 sensory cones along median furrow (Fig. 6C).

***Mandible*:** Basal joint massive, undivided (Figs 1D, E, 2C). Apical joint (mandibular gnathal lobe) damaged during preparation, but with long slender external tooth and a long condylus. Inner tooth 4-combed. Seven rows of pectinate lamellae, large intermediate area covered by scale-like spines. Additional intermediate area located at a lower level next to molar plate with more elongated spine-plates. Molar plate plane, without indentations or grooves, margins entirely surrounded by thick membranous fringes (Fig. 6D).

***Trunk*:** Collum (tergite 1) wider than head, shape similar to following tergites (Fig. 1A).

Tergites 2–19 (20) very thin, difficult to distinguish from one another. Width from tergite 1 to 8 gradually increasing, then decreasing to last tergite (Figs 1A, B, 7A). Each tergite bearing 6–8 longitudinal striae. Distances between striae increasing towards posterior margin. Surface of tergites with sparse isolated setae (Fig. 7A, B). Tergite 11 onwards posterior-lateral edge pronounced into a sharp-edged tip, becoming a longer and spine-like process at tergite 17. Limbus (posterior margin of tergite) smooth, without any structures. Endotergum (underside of posterior margin of tergite) smooth area without any specific structures (Fig. 7C). Posterior margin of tergite 19 and 20 with several short teeth (Fig. 7D)

Pleurites of rectangular shape, posterior margin overlapping first third of subsequent pleurite (Fig. 7B), wider than long. Pleurites similar to one another, but last pleurite on posterior margin with a well-rounded central indentation, providing space for the movements of the last leg.

***Legs*:** In all except first coxae fused with stigmatic plates (typical for Glomeridesmida, Wesener et al. 2014) (Figs 3A–H, 4A–C). Starting at coxo-stigmatic plate 9 (male holotype) or 11 (female paratype), every second coxa up to pair 21 (male) or 25 (female) apically with an eversible coxal pouch posteriorly of prefemur (Fig. 4B).

Leg-pair 1 with a well-rounded elliptical sternite visible in oral view. Femur 1.5 × longer than wide, tarsus slender and elongated, >7 times longer than wide (Fig. 3A). Apical half of tarsus without any spines or setae, except 1 or 2 apical spines, claw and paronychium (Fig. 3A, B).

Leg 2 (male) femur 1.7, tarsus >10 × longer than wide (Fig. 3C). Tarsus in apical half without any spines or setae except for single apical spine, claw and paronychium (Fig. 3D).

Legs 3–32 similar to first legs, e.g. midbody leg femur 1.8, tarsus >7 times longer than wide. Tarsus with 1 or 2 apical spines, claw and paronychium (Fig. 3D).

Penultimate leg with coxosternite narrow, with stigma opening and sternal part located below (instead of lateral to) of coxal part. Femur slightly longer than prefemur, 1.3 × longer than wide, tarsus shorter, >7 × longer than wide, apically with claw, apical spine and paronychium (Fig. 3E, F).

Ultimate leg pair (sensory leg) modified, consisting of a free large sternite, fused coxa-stigmatic plates plus 3 podomeres (Fig. 3G). First podomere with a conspicuous mesal bend. Last two podomeres extending postero-laterally (confused with telopod by pre-1941 authors). Podomere 3 apically with a large, long spine, longer than the podomere (Fig. 3H). Both legs widely separated from one another, connected by large sternite. Coxa-stigmatic plates large, almost as long as all 3 podomeres combined (Fig. 3G).

***Anal shield*:** glabrous, with a well-rounded edge. Subanal plate located behind last pair of legs, large and hyaline.

***Sexual characters*: Female**: second coxae on posterior side with prominent ovipositors protruding back to leg pair 5. Coxa protruding mesally as a short lobe. Ovipositors basally supported by an undivided plate (sternite?) (Fig. 4C). Ovipositor with a basal part consisting of 21 or 22 segments of (eversible?) half-rings, each carrying isolated setae in a regular distance to each other. Half rings anteriorly and posteriorly interrupted by two different, much slender tubes, also consisting of half-rings, running along the whole length of the ovipositor. Apical part of ovipositor with opening surrounded by four larger plates, each arising out of a tube of rings. All four plates covered by longer setae (Fig. 4D).

**Male**: second coxa with gonopore located mesally (Fig. 3C). Gonopore flanked by two sclerotized, plates resembling an open bivalve shell. Apical part of both plates with 2 or 3 longer setae (Fig. 5C).

***Male telopod*** consisting of syncoxite with inner horns and 4 podomeres (Figs 1C, 5A, D). Syncoxite covering basal podomeres laterally and in anterior view, medially rising into a smooth glabrous process. Process apically with two long inner horns; horns basally fused, completely separating more apically, diverging and running parallel to one another. Each horn apically slightly widening and spoon-shaped. Whole surface of horns sparsely covered by isolated, minute setae (Fig. 5D).

Telopod podomere 1 largest and most massive.

Podomere 2 wide, rectangular, apically with a large membranous area and mesally with a long and slender process. Membranous area well rounded, consisting of several inverse membranous folds. Slender process strongly elongated, as long as podomere 2, protruding between movable and immovable finger, inner surface excavated (Fig. 5A, B).

Podomere 3 long and slender, only 1/3 of width of podomere 2, inserting laterally on podomere 2. Posterior-lateral aspect of podomere 3 in apical part with slender, finger-shaped and well-rounded process called immobile finger, which makes up ca. half the length of podomere 3, inner surface of immobile finger excavated. Length of podomere 3, without immobile finger, 2 times longer than wide, with few isolated setae at margins in apical half (Fig. 5A, B). Immobile finger protruding up to 3/4 of length of podomere 4, called mobile finger (Fig. 5A, B).

Mobile finger (podomere 4), articulated to podomere 3 at base of immovable finger (Fig. 5A, B), slender and glabrous, 5 × longer than wide, posteriorly with an excavated area; at apex with a single long spine reaching 2/3 of length of immobile finger

###### Immature male.

The immature male has the same number of tergites, but one walking leg pair less than the mature male. The immature male is almost 1/3 shorter than the mature male. Coxal pouches starting at leg pair 9 as in mature male. Last pleurite with small well-rounded indentation at posterior margin. Telopod only slightly different from mature male: process of podomere 2 shorter, podomere 3 and 4 slenderer, immobile finger almost protruding up to apical end of movable finger.

###### Ecology.

**Figure 8.**
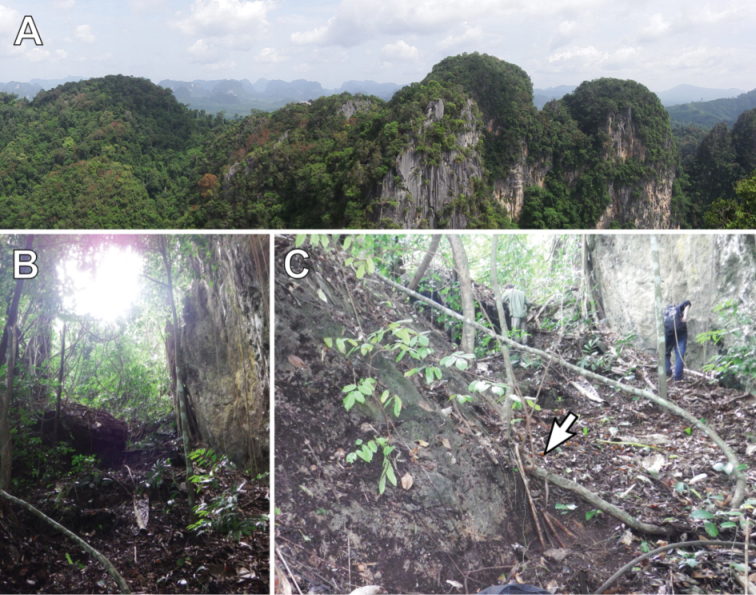
Habitat and type locality of *Glomeridesmus siamensis* sp. nov. **A** karstic landscape around Krabi Town, view from Tiger Cave temple (Wat Tham Suea) **B, C** slope underneath Tiger Cave temple at which *Glomeridesmus siamensis* sp. nov. was found, arrow indicates the overgrown rock from which the specimens were collected.

All specimens were collected by hand in the morning during rainy season in Krabi province, the second reported area for Glomeridesmida in Thailand (the first in Shelley 2011). They were found in a karst landscape covered with evergreen forest (Fig. 8A, B) in soils at a depth of 10–20 mm near horizontal roots on a partially overgrown rock. Despite numerous attempts, no *Glomeridesmus* was discovered outside the less than 30×30 centimeter of habitat, showing that their distribution might be dependent on some unknown microhabitat requirements. The habitat was a flat area on a western aspect (Fig. 8C). Average air temperature and relative humidity during observation was approximately 29 °C and 85%, respectively. The specimens were fast moving (at least as fast as Chordeumatida), capable of bending their flexible body 180° to quickly change directions. Furthermore, the male holotype carried an ‘amphoromorph’ fungus on one of its legs (Fig. 3B).

###### Additional locality data of unidentified Glomeridesmus from Thailand.

2 imm. F; **NHMD**; Chiang Mai Province, Doi Suthep N. P.; Konthathan (Montha Than waterfall?); 600–700 m; leg. NHMD, 30.ix.1981. 1 imm. F.; **NHMD**; Chiang Mai Province, Doi Suthep N. P.; Montha Than; 600–700 m; leg. NHMD, 26.ix.1981. 1 juv.; **NHMD**; Doi Suthep, 1150 m; Lok.3a; 29/9.1958; Birgit Degerbøl leg. Zool. Mus. Kbh. 1/7 59. Doi Suthep, 11–1200 m. 1 juv. (16 pairs of legs); **NHMD**; Doi Suthep, **No.** Kasens DSal; 14/9.1959, Birgit Degerbøl leg., Zool. Mus. Kbh. 1/7 59. 1 mature F (with nemertini parasite); **NHMD**; Doi Suthep, No. sigteprøve (= sifting sample) 3a?; 2/8.1958, Birgit Degerbøl leg., Zool. Mus. Kbh. 1/7 59. 1 juv. (8 pairs of legs); **NHMD**; Doi Suthep, 1150 m; **No.** Lok.3a; 29/9.1958; Birgit Degerbøl leg., Zool. Mus. Kbh. 1/7 59.

## Discussion

### Taxonomic characters in the Glomeridesmida

Descriptions of *Glomeridesmus* species were previously based on dubious somatic characters, i.e. species were most often separated by head morphology, body size, coloration and geography alone (e.g. Loomis 1964). The apparent paucity of males in *Glomeridesmus* populations makes precise taxonomic descriptions and comparisons across taxa challenging. Aside from one study of *G. marmoreus* Pocock, 1894 from Guadeloupe (Mauriès 1980), the intraspecific variation of taxonomic characters has not been evaluated due to the lack of material. Males were not known before 1936 (Loomis) and not thoroughly described until later (Carl 1941). Our sample also includes only a single mature male and a single immature male. Numerous *Glomeridesmus* species were described from female material only. Only nine of the 30 described species include a description of the telopods, which provides genus- and often species-specific characters in the closely related orders Glomerida and Sphaerotheriida. A comparison of the telopods of the nine known species with those of *G. siamensis* sp. nov., described here, highlights the usefulness of telopods as taxonomic characters. No future *Glomeridesmus* species should be described without male specimens and a telopod illustration. In doing so, researchers should be aware that the telopods of smaller males are difficult to distinguish from those of larger males. Unlike in the Glomerida and Sphaerotheriida, the telopods seem to lack characters identifying them as belonging to immature males. Considering *G. siamensis* sp. nov. the telopod of the smaller male has a relatively shorter projection on podomere 2, the unique character of *G. siamensis*. It is therefore currently entirely possible to accidentally describe the telopods of a smaller, potentially immature, male of a *Glomeridesmus* as those of a different species.

Contrary to the male sexual characters, the female ovipositors, while prominent, do not seem to carry much valuable information. The ovipositors of the Brazilian *G. spelaeus* Iniesta & Wesener, 2012 and of the Indian *G. indus* Carl, 1942 differ only slightly in the number of segments from *G. siamensis* sp. nov., for which the intraspecific variability is unknown, and which are difficult to count as basal parts are hidden.

Besides sexual characters, somatic characters also need to be assessed for their taxonomic importance. For example, it has been shown for the Glomerida and Sphaerotheriida that the endotergum, the legs and the antennae can carry relevant characters (Wesener 2016; Oeyen and Wesener 2018).

For the Glomeridesmida, the presence or absence of coxal pouches (Fig. 4B), as well as on which leg pair they start, seem to be important characters. Specimens must be well preserved to make this determination.

The modified ultimate leg-pair, the sensory legs, seem to contain few taxonomically important characters, but more comparisons between different species are necessary to evaluate this character. At least *G. siamensis* sp. nov. differs significantly in the shape of the sensory leg from other species of the genus, especially by the unusual shape of podomere 1 (Fig. 3G, H).

Surprisingly, the lamellae linguales of the gnathochilarium seem to carry taxonomically important characters in the Glomeridesmidae, as was discovered recently by Mauriès (2020). The lamellae linguales can be completely fused, both with one another and with the mentum (as in *G. spelaeus* from Brazil), divided from one another as well as from the mentum by sutures (as in *Glomeridesmoides* Mauriès, 2019) or fused with one another but separated from the mentum by a suture as in *G. siamensis* sp. nov. (Fig. 6A).

### Glomeridesmida diversity in Southeast Asia

Based on our findings and the insights from a Myriapoda inventory of a rainforest in Java (Hilgert et al. 2019), we suggest that the Glomeridesmida are far more diverse in Southeast Asia than previously known and are often overlooked when collecting by hand. This under sampling might result from their ability to move quickly to evade collection or their small size and inconspicuous appearance, especially compared to their larger relatives, the conspicuous pill millipedes (Glomerida) and giant pill-millipedes (Sphaerotheriida). Another possibility is that they are confined to micro-habitats. We found *Glomeridesmus siamensis* sp. nov. only on a single ca. 30×30 cm spot in a karst environment, which is known to provide micro-habitats and to show a great degree of endemism (Clements et al. 2006). The description of specimens faces two further challenges: the lack of mature male individuals, including among existing collections, and the low number of taxonomic experts for the group.

Only two species of Glomeridesmidae from Southeast Asia were described previously, *G. sumatranus* Pocock, 1894 from Sumatra and *G. javanicus* Attems, 1907 from Java; both are only known from female specimens. *G. sumatranus* was assigned to the genus *Zephroniodesmus* Pocock, 1894, which was later synonymized with *Glomeridesmus* by Loomis (1936) and Carl (1942). For *G. javanicus*, Verhoeff (1929) erected the genus *Javadesmus* Verhoeff, 1929, which was subsequently synonymized with *Glomeridesmus* by Carl (1942). Therefore, *Javadesmus* and *Zephroniodesmus* are available names that could be assigned to the Southeast Asian Glomeridesmidae. As the type species of both *Javadesmus* and *Zephroniodesmus* are only known from insufficiently described female specimens, we refrain from assigning *G. siamensis* sp. nov. to one of these genera. A study of male topotypic material is needed to clarify the affinities of the Southeast Asian *Glomeridesmus* species. The male sexual characters of *G. siamensis* sp. nov. are strikingly different from those of the South American and Indian species. Several representatives of Glomeridesmida from throughout Southeast Asia, including Sumatra, are currently under study.

## Supplementary Material

XML Treatment for Zephroniodesmidae

XML Treatment for Glomeridesmus

XML Treatment for Glomeridesmus siamensis
